# Fossil evidence of evolutionary convergence in juvenile dental morphology and upper canine replacement in sabertooth carnivores

**DOI:** 10.1002/ece3.5732

**Published:** 2019-10-23

**Authors:** Matthew Aleksander Wysocki

**Affiliations:** ^1^ Department of Pathology and Anatomical Sciences Jacobs School of Medicine and Biomedical Sciences University at Buffalo Buffalo NY USA

**Keywords:** dental morphology, mammals, ontogeny, tooth development

## Abstract

The convergent suite of morphological traits characterizing the mammalian sabertooth ecomorphology is well documented, including modifications of the dental and osteological portions of the masticatory apparatus from a less‐specialized carnivore condition. Equally important is how those specialized adult morphologies developed through ontogeny because previous studies have shown that growing such specialized craniodental traits may require evolutionary modification of growth patterns and tooth replacement mechanisms. Despite the understanding of convergent morphological specialization in adult sabertooth carnivores, the possibility of a convergent ontogenetic trajectory toward those adult morphologies has not been rigorously examined. The present study examines numerous previously undescribed juvenile nimravid specimens. The results provide insights about nimravid ontogeny and show, for the first time, that the nimravid sabertooth lineage included species in which the permanent upper canine erupted within a lingual concavity of the deciduous upper canine until it reached comparable crown height beyond the alveolar border. Furthermore, this investigation assesses the juvenile morphology and upper canine replacement of felid and barbourofelid sabertooth taxa. The results provide evidence of convergence in deciduous upper canine morphology of three sabertooth carnivore lineages (i.e., nimravid, felid, and barbourofelid), as well as preliminary evidence of convergence in the upper canine replacement process. It might be beneficial for studies of extreme morphological specialization to simultaneously consider convergence in adult morphologies and how morphologies change through ontogeny.

## Graphical abstract

The convergent suite of morphological traits characterizing the mammalian sabertooth ecomorphology is well documented, including modifications of the dental and osteological portions of the masticatory apparatus. Equally important is how those specialized adult morphologies developed through ontogeny. All in all, the results of this investigation provide evidence that three sabertooth carnivore lineages (i.e., nimravid, felid, and barbourofelid) converged in both their deciduous upper canine morphology and upper canine replacement process, which suggests that studies of extreme morphological specialization should simultaneously consider convergence in adult morphologies and how morphology changes through ontogeny.

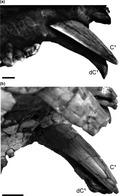

## 1 INTRODUCTION

Sabertooth carnivores are terrestrial vertebrate taxa that possess high‐crowned, mediolaterally compressed upper canine teeth (Antón, 2013; Martin, 1980; Meachen‐Samuels, 2012). This conspicuous dentition is most commonly discussed regarding the Pleistocene species, *Smilodon fatalis*, but saberteeth occur in multiple evolutionary lineages (i.e., felid, nimravid, barbourofelid, thylacosmilid, creodont, and gorgonopsid; Antón, 2013; Schultz, Schultz, & Martin, 1970; Wysocki & Feranec, 2018). Because this idiosyncratic morphological structure is not present in any extant carnivorous terrestrial species, there have been many questions about how sabertooth carnivores used their teeth and how they lived within their respective ecosystems (Antón, 2013; Cope, 1880; Gonyea, 1976; McDonald, 2018). Much of the research has centered around the adult morphological specializations and how the permanent saberteeth functioned, but many questions still remain about the ontogeny and evolution of sabertooth carnivores (McHenry, Wroe, Clausen, Moreno, & Cunningham, 2007; Rawn‐Schatzinger, 1983; Tejada‐Flores & Shaw, 1984; Wroe et al., 2013). For instance, although evolutionary convergence has been documented in the adult form of sabertooth carnivores, the question of whether or not sabertooth carnivores of multiple lineages had similar upper canine replacement processes and morphological specializations in their juvenile forms has not been examined (Antón, 2013).

The classic study of tooth eruption in *S. fatalis* (Felidae) by Tejada‐Flores and Shaw (1984) ascertained important insights about the tooth development and ecology of this sabertooth species, including that the deciduous upper canine remains in place until the permanent upper canine erupts along its lingual surface to approximately the same crown height beyond the alveolar border. The recent description of the ontogenetically youngest known specimen of *S. fatalis* (LACM 124681) shows the upper canine alveolus containing the permanent upper canine growing adjacent to the partially formed deciduous upper canine, thereby suggesting that this tooth replacement process began early in development for this species (Shaw & Quinn, 2015). Examination of another ontogenetic series of juvenile specimens yielded key findings about the tooth development of *Barbourofelis* (Barbourofelidae), as well as implications for the juvenile behavioral ecology of this genus (Bryant, 1988, 1990). The current investigation provides new data about the upper canine replacement process of sabertooth carnivores of Nimravidae, an older sabertooth lineage with the earliest taxa occurring in the Late Eocene (Antón, 2013; McKenna & Bell, 1997). Furthermore, this investigation evaluates whether or not the juvenile morphologies and upper canine replacement processes are similar across the felid, barbourofelid, and nimravid sabertooth lineages in order to contribute to the understanding of how sabertooth carnivores evolved and developed their hypertrophied upper canine dentition.

## 2 MATERIALS AND METHODS

The juvenile morphologies and upper canine replacement processes of sabertooth taxa of the Nimravidae (Cope, 1880), Felidae, and Barbourofelidae (Morlo, Peigne, & Nagel, 2004) were studied through the examination of 15 juvenile sabertooth specimens from the AMNH and UCMP. In particular, 11 previously undescribed juvenile nimravid specimens were analyzed to determine the upper canine replacement process of the nimravid sabertooth lineage. The morphology and development of two juvenile specimens of the felid *S. fatalis* were also examined. In addition, the current study included a detailed reexamination of the tooth development of two previously described juvenile specimens of the barbourofelid, *Barbourofelis morrisi* (Bryant, 1988, 1990; Schultz et al., 1970). As the focus of this investigation is on developmental and evolutionary processes, species‐level assignments and reassessments of this fossil material are beyond the scope of this investigation.

### 2.1 Institutional abbreviations

AMNH FM: Frick Fossil Mammals Collection, Division of Paleontology, American Museum of Natural History, New York, NY, USA; LACM HC: Hancock Collection, Natural History Museum of Los Angeles County, Los Angeles, CA, USA; UCMP: University of California Museum of Paleontology, Berkeley, CA, USA; UF: Florida Museum of Natural History, University of Florida, Gainesville, FL, USA.

## 3 RESULTS

### 3.1 AMNH FM 39100 (Nimravidae)

Description: Nearly complete cranium with the majority of the permanent dentition already erupted. Although fragmentary, the I^1^, I^2^, and I^3^ appear to be fully erupted given the condition of the incisor roots and alveoli. The right and left P^2^, P^3^, P^4^, and M^1^ are all present. On the right side, the C^1^ has an erupted length of 5 mm and occupies the lingual concavity of the dC^1^. On the left side, the C^1^ has erupted beyond the alveolar border by about 1 mm and is positioned on the lingual side of the dC^1^ alveolus; the left dC^1^ does not appear to have been shed during the life of the individual. Table 1 provides a more concise description of AMNH FM 39100 and the specimens mentioned below, with an emphasis on the dental morphology and upper canine replacement processes of these sabertooth taxa.

**Table 1:** Summary of juvenile dentition

Specimen	Dental condition
AMNH FM 39100 Nimravidae	I^1^, I^2^, I^3^ fully erupted. P^2^, P^3^, P^4^, M^1^ partially erupted. Right C^1^ erupted length beyond the alveolar border = 5 mm; right C^1^ occupies the lingual concavity of the right dC^1^. Left C^1^ erupted length beyond the alveolar border = 1 mm; left C^1^ positioned on the lingual side of the dC^1^ alveolus; left dC^1^ does not appear to have been shed during the life of the individual
AMNH FM 62081 Nimravidae	I^3^, P^3^, P^4^, M^1^, dC^1^ present. Both left C^1^ and right C^1^ erupted lengths beyond the alveolar border = 4 mm. Each C^1^ positioned directly lingual to the respective dC^1^
AMNH FM 62070 Nimravidae	I^1^, I^2^, I^3^, P^2^, P^3^, P^4^, M^1^, dC^1^ fully erupted. C^1^ erupted lengths beyond the alveolar border = 5 mm. C^1^ immediately lingual to dC^1^
AMNH FM 62041 Nimravidae	I^3^, P^3^, P^4^, M^1^, dC^1^ present. Natural cross sections of the right dC^1^ and right C^1^ show that the C^1^ is within the lingual concavity of the dC^1^. Left C^1^ erupted length beyond the alveolar border = 10 mm; left C^1^ erupting within the lingual concavity of the left dC^1^
AMNH FM 125658 Nimravidae	I^1^ alveolus, I^2^, I^3^ roots; all upper incisors likely fully erupted. P^3^, P^4^, M^1^, dC^1^ present. Left C^1^ erupted length beyond the alveolar border = 12 mm; distal end of right C^1^ crown missing. Each C^1^ is positioned within the lingual concavity of the respective dC^1^
AMNH FM 62110 Nimravidae	I^1^, I^2^, I^3^, P^3^, P^4^, M^1^, dC^1^ erupted; P^2^ alveolus is present. Fractured C^1^ are partially erupted and positioned directly lingual to the dC^1^
AMNH FM 62111 Nimravidae	I^1^, I^2^, I^3^, P^2^, P^3^, P^4^, M^1^ fully erupted. dC^1^ are erupted. Fractured left dC^1^ with partially erupted left C^1^ along its lingual surface
AMNH FM 69427 Nimravidae	I^3^, P^3^, P^4^, M^1^, dC^1^ fully erupted. Well‐developed right C^1^ directly lingual to the right dC^1^
AMNH FM 62013 Nimravidae	I^1^, I^2^, I^3^, P^2^, P^3^, P^4^, M^1^, dC^1^ erupted. Both right dC^1^ and right C^1^ are fractured at the alveolar border; the partially erupted C^1^ is positioned immediately adjacent to the dC^1^ within its lingual concavity
AMNH FM 125675 Nimravidae	I^1^, I^2^, I^3^, P^3^, P^4^, M^1^, dC^1^ fully erupted. C^1^ erupted lengths beyond the alveolar border = over 35 mm. C^1^ are positioned immediately lingual to the dC^1^
AMNH FM 69421 Nimravidae	I^1^, I^2^, I^3^, P^2^, P^3^, P^4^, M^1^, dC^1^ fully erupted. Left C^1^ erupted length beyond the alveolar border = 40 mm; left C^1^ is located directly lingual to the left dC^1^. Right C^1^ exhibits a similar stage of eruption; tip of right C^1^ crown is fractured. Right C^1^ located immediately lingual to the right dC^1^
UCMP 152565 Felidae (*Smilodon fatalis*)	dP^3^, dP^4^, dC^1^ erupted. C^1^ is about to erupt and it is positioned within the center of the lingual concavity of the dC^1^
UCMP 152566 Felidae (*Smilodon fatalis*)	dC^1^ erupted. C^1^ has started to erupt beyond the alveolar border and it is positioned within the center of the lingual concavity of the dC^1^
AMNH FM 61895 Barbourofelidae (*Barbourofelis morrisi*)	I^1^, I^2^, I^3^, P^3^, P^4^ erupted. M^1^ alveolus present. Left dC^1^ erupted just beyond the alveolar border; left C^1^ is unerupted. Proximal portions of the right dC^1^ and right C^1^; right C^1^ is centered along the lingual surface of the right dC^1^. dC^1^ and C^1^ display nearly parallel orientation to one another
AMNH FM 79999 Barbourofelidae (*Barbourofelis morrisi*)	I^1^, I^2^, I^3^, P^3^, P^4^, M^1^ erupted. Erupted sabers are definitely dC^1^; the left C^1^ is about to erupt and is positioned immediately lingual to the left dC^1^

Geological Age: Orellan.

Locality Notes: Scenic, Pennington County, SD, USA; Oreodon.

Collection Date: 1940.

### 3.2 AMNH FM 62081 (Nimravidae)

Description: Partial cranium with an intact left I^3^, upper canines (i.e., dC^1^ and C^1^), as well as right and left P^3^, P^4^, and M^1^. Both the left C^1^ and right C^1^ have erupted to about 4 mm beyond the alveolar border, and each is positioned directly lingual to the respective dC^1^.

Geological Age: Oligocene.

Locality Notes: WY, USA.

Collection Date: 1938.

### 3.3 AMNH FM 62070 (Nimravidae)

Description: Nearly complete cranium except for a missing left zygomatic arch. The right and left I^1^, I^2^, I^3^, P^2^, P^3^, P^4^, M^1^, and dC^1^ have erupted. The partially erupted C^1^ (i.e., erupted lengths of approximately 5 mm) are immediately lingual to the dC^1^.

Geological Age: Orellan.

Locality Notes: 2.5 Miles North of Chadron, NE, USA; Lower part of Orella.

Collection Date: 1944.

### 3.4 AMNH FM 62041 (Nimravidae)

Description: Right and left maxillae. The right maxilla includes the dC^1^, I^3^, P^3^, P^4^, and C^1^. The dC^1^ is partially erupted. Natural cross sections of right dC^1^ and right C^1^ show that the C^1^ is situated within the lingual concavity of the dC^1^. The left maxilla includes the dC^1^, P^3^, P^4^, M^1^, and C^1^. The left dC^1^ and left C^1^ are nearly complete and also show the C^1^ erupting within the lingual concavity of the dC^1^. The left C^1^ has an erupted length of 10 mm beyond the alveolar border.

Geological Age: Orellan.

Locality Notes: 3 miles Southeast of Scenic, Pennington County, SD, USA; Lower Oreodon.

Collection Date: 1945.

### 3.5 AMNH FM 125658 (Nimravidae)

**Figure 1:**
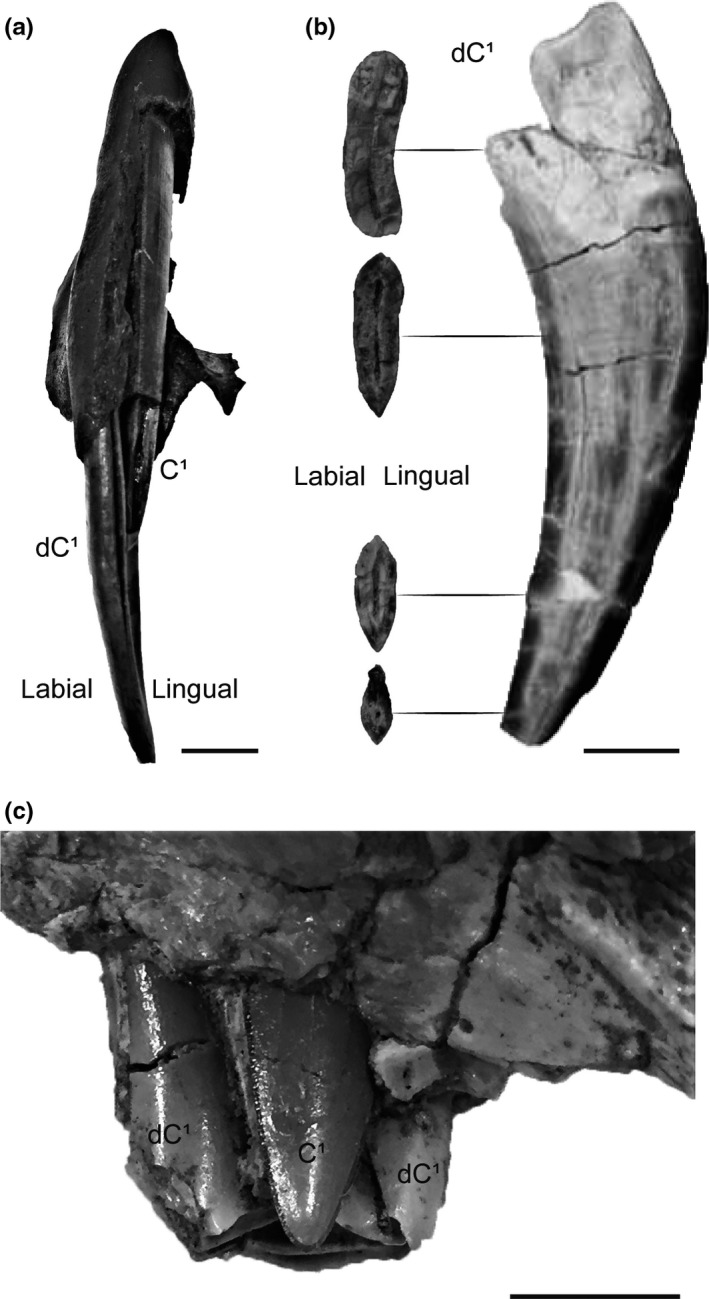
Lingual concavity of the deciduous upper canine of three sabertooth carnivore lineages. (a) Felid, UCMP 152566 (*Smilodon fatalis*). Right permanent upper canine (C^1^) erupting within the lingual concavity of the right deciduous upper canine (dC^1^), anterior view. (b) Barbourofelid, LACM 154061 (*Barbourofelis whitfordi*). Right deciduous upper canine (dC^1^) with cross‐sectional photographs of the natural breaks that show the lingual concavity of this tooth. Modified from Tseng, Takeuchi, and Wang (2010). (c) Nimravid, AMNH FM 125658. Left permanent upper canine (C^1^) erupting within the lingual concavity of the left deciduous upper canine (dC^1^), lingual view. Scale bars = 1 cm

Description: Partial cranium with right and left dC^1^, P^3^, P^4^, M^1^, and C^1^. The right and left I^2^ and I^3^ roots are present, as well as the left I^1^ alveolus, suggesting that all of the upper incisors were fully erupted. The C^1^ are partially erupted and positioned within the lingual concavities of the dC^1^ (Figure 1). The distal end of the right C^1^ crown is missing, whereas the left C^1^ is complete and has an erupted length of 12 mm beyond the alveolar border.

Geological Age: Early Chadronian to Late Orellan.

Locality Notes: Plunkett‐Parsons, Sioux County, NE, USA; 5′ below Middle Banded Layer.

Collection Date: 1964.

### 3.6 AMNH FM 62110 (Nimravidae)

Description: Partial cranium with nearly complete dentition. The left I^1^, I^2^, I^3^, P^3^, P^4^, M^1^, and dC^1^ are erupted, and P^2^ alveolus is present. The right I^1^ alveolus is present and the I^2^, I^3^, P^3^, P^4^, M^1^, and dC^1^ are all erupted. The fractured C^1^ are partially erupted and directly lingual to the dC^1^.

Geological Age: Early Chadronian to Late Orellan.

Locality Notes: Plunkett Ranch, Hat Creek Basin, NE, USA.

Collection Date: 1944.

### 3.7 AMNH FM 62111 (Nimravidae)

Description: Partial cranium and mandible. The permanent upper dentition (I^1^, I^2^, I^3^, P^2^, P^3^, P^4^, M^1^) has fully erupted except for the C^1^, and the permanent lower dentition has completely erupted. The dC^1^ are still present. The fractured left dC^1^ reveals the partially erupted left C^1^ along its lingual surface.

Geological Age: Early Chadronian to Late Orellan.

Locality Notes: Warbonnet Cr., USA.

Collection Date: 1944.

### 3.8 AMNH FM 69427 (Nimravidae)

Description: Fragmentary cranium with fully erupted right dC^1^, I^3^, P^4^, M^1^, as well as completely erupted left P^3^, P^4^, and M^1^. A well‐developed right C^1^ occupies the space directly lingual to the right dC^1^.

Geological Age: Chadronian.

Locality Notes: Niobrara County, WY, USA; White River, Chadron.

### 3.9 AMNH FM 62013 (Nimravidae)

Description: A partial cranium with the right side showing the dC^1^, I^1^, I^2^, I^3^, C^1^, P^2^, P^3^, P^4^, and M^1^. The damaged left side of the cranium possesses the P^3^ and P^4^. Both the right dC^1^ and right C^1^ are fractured at the alveolar border. The partially erupted C^1^ is positioned immediately adjacent to the dC^1^ within the lingual concavity.

Geological Age: Orellan.

Locality Notes: 1 Mile South of Cottonwood Pass, Shannon County, SD, USA; Lower Oreodon.

Collection Date: 1938.

### 3.10 AMNH FM 125675 (Nimravidae)

Description: Mostly complete cranium with entirely erupted right and left dC^1^, I^1^, I^2^, I^3^, P^3^, P^4^, and M^1^. The partially erupted C^1^ have erupted lengths of more than 35 mm beyond the alveolar border. The C^1^ are positioned immediately lingual to the dC^1^.

Geological Age: Early Chadronian to Late Orellan.

Locality Notes: West of Anthill area 85′ above P.W., Niobrara County, WY, USA.

Collection Date: 1963.

### 3.11 AMNH FM 69421 (Nimravidae)

Description: Fragmentary cranium with left dC^1^, I^1^, I^2^, I^3^, C^1^, P^2^, P^3^, P^4^, M^1^ and the right I^1^, I^3^, C^1^, P^2^, P^3^, P^4^, and M^1^. On the left side, the C^1^ is located directly lingual to the dC^1^; the C^1^ has erupted approximately 40 mm beyond the alveolar border. The right C^1^ exhibits a similar stage of eruption, although the tip of the right C^1^ crown is fractured; right C^1^ is located immediately lingual to the right dC^1^ root.

Geological Age: Early Chadronian to Late Orellan.

Locality Notes: 1 Mile West of R. Thompsons, USA; 80′ above PWL.

### 3.12 UCMP 152565 *Smilodon fatalis* (Felidae)

Description: Right maxilla with dC^1^, dP^3^, dP^4^ erupted. The C^1^ is about to erupt, and it is positioned within the center of the lingual concavity of the dC^1^. The dC^1^ to C^1^ orientation demonstrates the close proximity of these sabers to one another, as well as the large amount of surface area overlap.

Geological Age: Pleistocene.

Locality Notes: LACM locality 3874, Rancho La Brea, Los Angeles, CA, USA.

### 3.13 UCMP 152566 *Smilodon fatalis* (Felidae)

**Figure 2:**
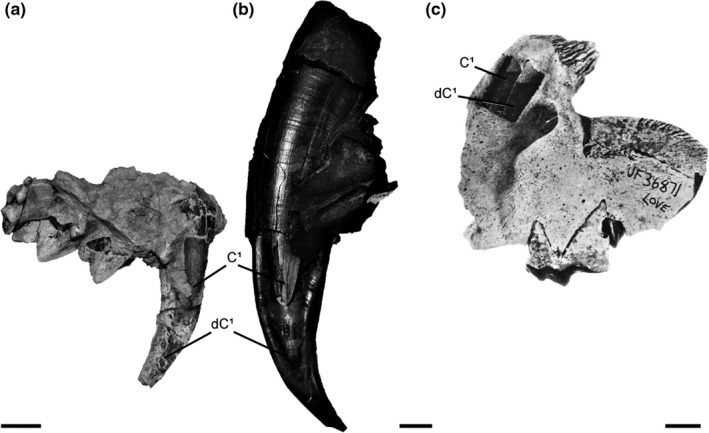
Upper canine replacement process of three sabertooth carnivore lineages. (a) Left maxilla of nimravid, AMNH FM 62041, showing the permanent upper canine (C^1^) erupting in the lingual concavity of the deciduous upper canine (dC^1^), lingual view. (b) Right maxilla of felid, UCMP 152566 (*Smilodon fatalis*), showing the permanent upper canine (C^1^) erupting in the lingual concavity of the deciduous upper canine (dC^1^), lingual view. (c) Left maxilla of barbourofelid, UF 36871 (*Barbourofelis loveorum*), showing the permanent upper canine (C^1^) erupting in the lingual concavity of the deciduous upper canine (dC^1^), buccal view. The upper canines are visible through a window that was cut into the maxilla. Modified from Bryant (1988). Scale bars = 1 cm

Description: Right maxilla showing the dC^1^ erupted. The C^1^ has started to erupt beyond the alveolar border, and it is positioned within the center of the lingual concavity of the dC^1^ (Figure 2). UCMP 152566, as well as the aforementioned UCMP 152565, represent ontogenetic stages that provide very clear examples of the juvenile morphology and upper canine replacement process of this felid sabertooth species. These results are consistent with findings from the analysis of an extensive ontogenetic series of *S. fatalis* specimens (Tejada‐Flores & Shaw, 1984).

Geological Age: Pleistocene.

Locality Notes: LACM locality 3874, Rancho La Brea, Los Angeles, CA, USA.

### 3.14 AMNH FM 61895 *Barbourofelis morrisi* (Barbourofelidae)

Description: Partial cranium with damage to the ventral surface of the right side. The left side shows the erupted I^1^, I^2^, I^3^, P^3^, and P^4^, as well as the M^1^ alveolus. Previous study of AMNH FM 61895 described the presence of a right permanent upper canine developing lingual to the deciduous upper canine (Bryant, 1990). Additional examination of the specimen reveals that the left dC^1^ has erupted just beyond the alveolar border, whereas the left C^1^ is still unerupted. The damage to the right side of the cranium exposes the proximal portions of the right dC^1^ and right C^1^. Despite the surrounding damage, the teeth are still in their natural positions with the right C^1^ centered along the lingual surface of the dC^1^. Furthermore, the dC^1^ and C^1^ display a nearly parallel orientation to one another.

Geological Age: Early Clarendonian.

Locality Notes: Machaerodus Quarry, Cherry County, NE, USA; Ogallala, Ash Hollow, Merritt Dam.

Collection Date: 1934.

### 3.15 AMNH FM 79999 *Barbourofelis morrisi* (Barbourofelidae)

**Figure 3:**
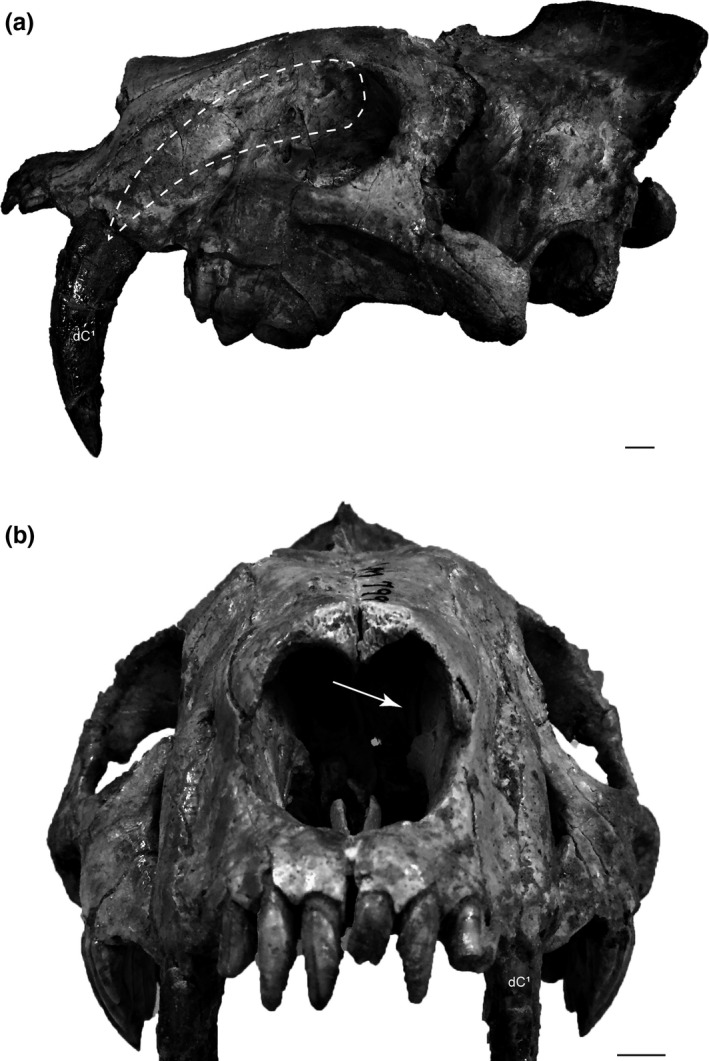
Growth and eruption of the permanent upper canine (C^1^) directly lingual to the deciduous upper canine (dC^1^) in the barbourofelid, *Barbourofelis morrisi*. (a) AMNH FM 79999 (*Barbourofelis morrisi*) exhibiting the erupted deciduous upper canine (dC^1^), lateral view. Dashed white line indicates the position of the well‐developed permanent upper canine (C^1^). (b) Close inspection of the natural fractures in AMNH FM 79999 (*Barbourofelis morrisi*) confirms the presence and location of the well‐developed permanent upper canine about to erupt directly lingual to the deciduous upper canine (white arrow indicates a location for observing the permanent upper canine). Scale bars = 1 cm

Description: The *B. morrisi* Holotype is a nearly complete juvenile skull with erupted I^1^, I^2^, I^3^, P^3^, P^4^, and M^1^. Bryant (1988) makes the exceptional deduction that the erupted sabers of AMNH FM 79999 are probably deciduous upper canines and that the maxillae likely contain permanent upper canines; concepts that are now confirmed. Close inspection of natural fractures in the specimen reveals that a large portion of the left C^1^ crown has formed, indicating that the erupted sabers are the dC^1^ (Figure 3). The left C^1^ is about to erupt and is positioned immediately lingual to the left dC^1^.

Geological Age: Early Hemphillian.

Locality Notes: Leptartcus Quarry, Cherry County, NE, USA; Ogallala, Ash Hollow.

Collection Date: 1936.

## 4 DISCUSSION

Previous studies of ontogenetic series provided key foundational insights about the tooth development and life history of sabertooth taxa (i.e., the felid *S. fatalis* and the barbourofelid *Barbourofelis*) (Bryant, 1988, 1990; Tejada‐Flores & Shaw, 1984), but little was known about how nimravid sabertooth carnivores replaced their upper canine dentition. Additionally, the question of whether or not taxa of the nimravid, felid, and barbourofelid sabertooth carnivore lineages had similar juvenile dental morphology and upper canine replacement processes remained unexamined. The results of the current investigation indicate that taxa of Nimravidae possess deciduous upper canine morphology that includes marked mediolateral compression and a concavity along the lingual surface. Analysis of this nimravid ontogenetic series also reveals that the deciduous upper canine is still in position when all of the permanent upper dentition, except for the permanent upper canine, finishes erupting. The permanent upper canine is observably the last tooth to begin its eruption process, which is consistent with the findings of Bryant (1988) regarding *Dinictis* (Nimravidae). The results of the current study also indicate that the location of initial permanent upper canine eruption is directly medial to the deciduous upper canine, within its lingual concavity. In addition, the results from analysis of these nimravid specimens show that the permanent upper canine continues to erupt within the lingual concavity of the deciduous upper canine and that the deciduous upper canine remains in position until the erupting permanent upper canine has reached approximately the same crown height beyond the alveolar border.

**Figure 4:**
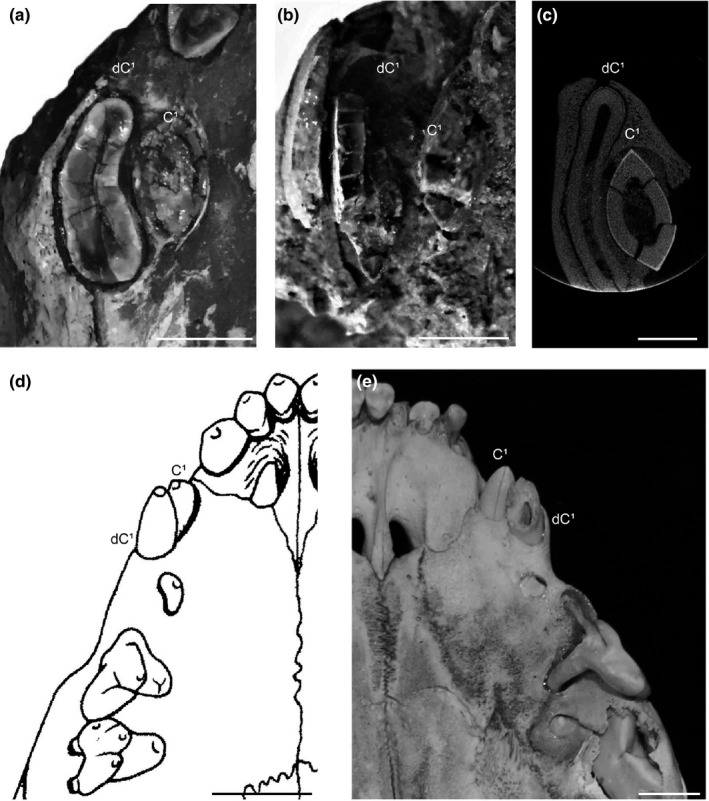
The upper canine morphology and replacement process of sabertooth taxa (above) versus the upper canine morphology and replacement process of closely related conical‐toothed taxa (below). The upper canine replacement process of three sabertooth lineages involves permanent upper canine (C^1^) eruption medial, within the lingual concavity of the deciduous upper canine (dC^1^). The upper canine replacement process of related conical‐toothed taxa consists of the permanent upper canine (C^1^) erupting anterior and medial to the deciduous upper canine (dC^1^). (a) Nimravid, AMNH FM 62013. Natural fractures of the upper canine teeth at the alveolar border showing cross sections of the right permanent upper canine (C^1^) erupting within the lingual concavity of the right deciduous upper canine (dC^1^), ventral view. (b) Barbourofelid, AMNH FM 61895 (*Barbourofelis morrisi*). Damaged maxilla and natural fractures of the upper canine teeth revealing the right permanent upper canine (C^1^) developing lingual to the right deciduous upper canine (dC^1^), anteroventral view. (c) Felid, UCMP 152566 (*Smilodon fatalis*). Micro‐CT slice of the upper canine teeth showing cross sections of the right permanent upper canine (C^1^) within the lingual concavity of the right deciduous upper canine (dC^1^), ventral view. Modified from Micro‐CT data Wysocki, Feranec, Tseng, and Bjornsson (2015). (d) Mustelid, USNM 222659 (*Taxidea taxus*). Right permanent upper canine (C^1^) erupting anterior and medial to the right deciduous upper canine (dC^1^), ventral view. Modified from Long (1974). (e) Felid, AMNH 17420 (*Panthera tigris*). Left permanent upper canine (C^1^) erupting anterior and medial to the left deciduous upper canine (dC^1^), ventral view. The natural fracture of the deciduous upper canine (dC^1^) demonstrates the conical morphology that lacks the marked lingual concavity and mediolateral compression found in the deciduous upper canines of the sabertooth taxa. Scale bars = 1 cm

This information about the ontogeny of taxa of Nimravidae makes it possible to assess juvenile morphologies and upper canine replacement processes across the nimravid, felid, and barbourofelid sabertooth lineages. It is evident that taxa of Nimravidae, Felidae, and Barbourofelidae exhibit similar deciduous upper canine morphology that includes considerable mediolateral compression as well as the presence of a concave lingual surface, suggesting that evolutionary convergence occurred in both the adult morphology and the juvenile morphology of three sabertooth lineages (Bryant, 1988; Tejada‐Flores & Shaw, 1984). Also, the upper canine replacement process of the nimravid specimens described above is very similar to the upper canine replacement processes that occur in taxa of Felidae (i.e., *S. fatalis*) and Barbourofelidae (i.e., *B. morrisi* and *Barbourofelis loveorum*). Particularly, the orientation of the permanent upper canine as it erupts directly medial, within the deciduous upper canine's lingual concavity is comparable in taxa of these three sabertooth lineages (Baskin, 1981; Bryant, 1988, 1990; Tejada‐Flores & Shaw, 1984), which might be indicative of evolutionary convergence in tooth development; however, a comprehensive review of the upper canine replacement processes of more sister taxa and the discoveries of additional fossils are necessary to thoroughly test this hypothesis. Preliminary supporting evidence is apparent in the upper canine replacement processes of related conical‐toothed taxa such as *Panthera tigris, Panthera leo, and Taxidea taxus* in which the permanent upper canine erupts in an anteromedial position relative to the deciduous upper canine, whereas the upper canine replacement process of these sabertooth taxa involves permanent upper canine eruption that is medial to the deciduous upper canine and centered within a lingual concavity on the deciduous upper canine (Long, 1974; Smuts, Anderson, & Austin, 1978) (Figure 4).

**Figure 5:**
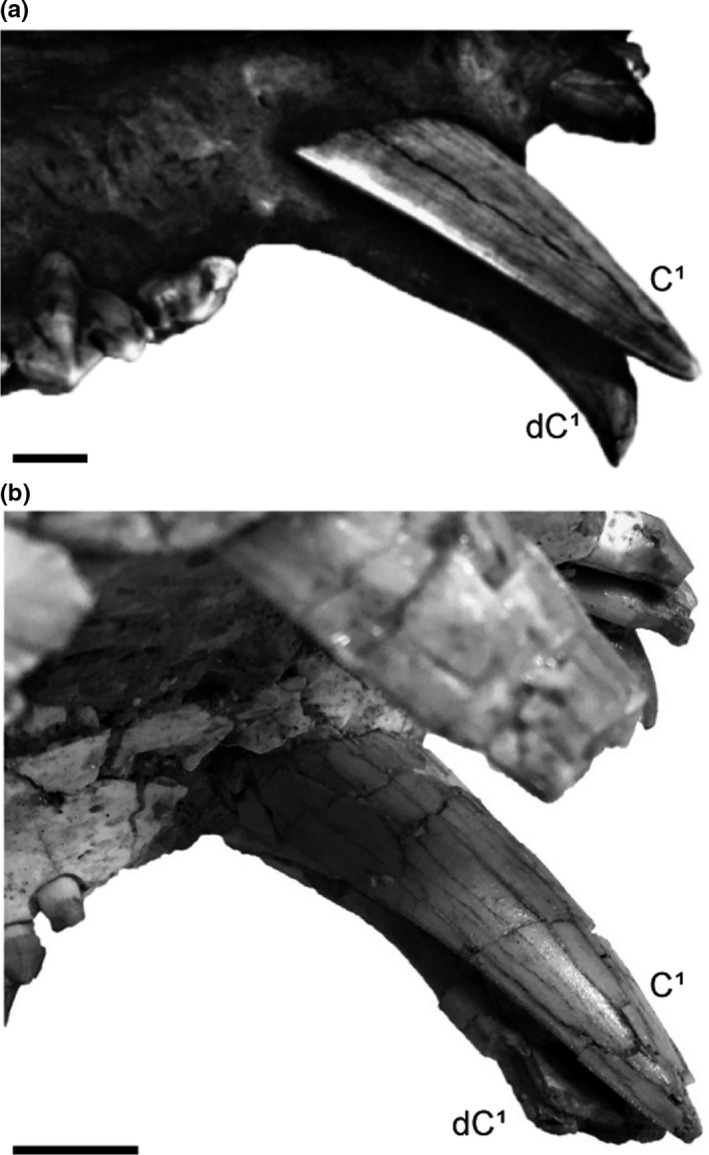
Late stage of the upper canine replacement process in these sabertooth taxa. (a) Felid, LACM 2001‐7 (*Smilodon fatalis*). Left permanent upper canine (C^1^) erupting lingual to the left deciduous upper canine (dC^1^), ventromedial view. Modified from Tejada‐Flores and Shaw (1984). (b) Nimravid, AMNH FM 69421. Left permanent upper canine (C^1^) erupting lingual to the left deciduous upper canine (dC^1^), ventromedial view. Scale bars = 1 cm

As there is no extant sabertooth carnivore, the manner in which the extremely specialized dentition of sabertooth carnivores developed and functioned throughout ontogeny has been of great interest and led to proposal of a variety of hypotheses concerning the juvenile behavioral ecology of multiple taxa, especially regarding hunting (Bryant, 1988, 1990; Rawn‐Schatzinger, 1981, 1983; Tejada‐Flores & Shaw, 1984). In particular, Tejada‐Flores and Shaw (1984) hypothesized that the upper canine replacement of *S. fatalis*, in which the deciduous upper canine remains in position as the permanent upper canine reaches a comparable erupted crown height, would have maintained functional capacity at this tooth position. Biomechanical studies are required in order to test this continuous functionality hypothesis and to determine the exact functional capabilities of juvenile sabertooth carnivores, but the results of the current study demonstrate that taxa of the nimravid lineage also had upper canine replacement in which upper canine presence is maintained. The similar juvenile morphology and developmental pattern of these nimravids and the felid *S. fatalis* are especially evident in AMNH FM 125675, AMNH FM 69421, and LACM 2001‐7 (Figure 5). All in all, the remarkable juvenile sabertooth specimens of Nimravidae, Felidae, and Barbourofelidae provide evidence of convergent evolution in the deciduous upper canine morphologies and preliminary evidence of possible convergent evolution in the upper canine replacement processes of three sabertooth lineages (Baskin, 1981; Bryant, 1988, 1990; Tejada‐Flores & Shaw, 1984). Although evolutionary studies commonly focus on the morphological specializations of adult individuals, it might be beneficial for explanatory models of extreme morphological specialization in vertebrates to concurrently consider convergence in adult morphologies and how those morphologies change through ontogeny.

## CONFLICT OF INTEREST

None declared.

## AUTHOR CONTRIBUTION

MAW conceived and designed the study, collected the data, analyzed the data, and wrote the manuscript.

## Data Availability

All data supporting these findings have been made available within the manuscript.

## References

[ece35732-bib-0001] Antón, M. (2013). Sabertooth. Bloomington, IN: Indiana University Press.

[ece35732-bib-0002] Baskin, J. A. (1981). *Barbourofelis* (Nimravidae) and *Nimravides* (Felidae), with a description of two new species from the late Miocene of Florida. Journal of Mammalogy, 62, 122–139.

[ece35732-bib-0003] Bryant, H. N. (1988). Delayed eruption of the deciduous upper canine in the sabertoothed carnivore *Barbourofelis lovei* (Carnivora, Nimravidae). Journal of Vertebrate Paleontology, 8, 295–306.

[ece35732-bib-0004] Bryant, H. N. (1990). Implications of the dental eruption sequence in *Barbourofelis* (Carnivora, Nimravidae) for the function of upper canines and the duration of parental care in sabretoothed carnivores. Journal of Zoology, 222, 585–590.

[ece35732-bib-0005] Cope, E. D. (1880). On the extinct cats of America. The American Naturalist, 14, 833–858.

[ece35732-bib-0006] Gonyea, W. J. (1976). Behavioral implications of saber‐toothed felid morphology. Paleobiology, 2, 332–342.

[ece35732-bib-0007] Long, C. A. (1974). Growth and development of the teeth and skull of the wild North American badger, *Taxidea taxus* . Transactions of the Kansas Academy of Science, 77, 106–120.

[ece35732-bib-0008] Martin, L. D. (1980). Functional morphology and the evolution of cats. Transactions of the Nebraska Academy of Sciences, VIII, 141–154.

[ece35732-bib-0009] McDonald, H. G. (2018). *Smilodon*: A short history of becoming the iconic sabertooth In Werdelin L., McDonald H. G. & Shaw C. A. (Eds.), Smilodon: The iconic sabertooth (pp. 1–13). Baltimore, Maryland: Johns Hopkins University Press.

[ece35732-bib-0010] McHenry, C. R. , Wroe, S. , Clausen, P. D. , Moreno, K. , & Cunningham, E. (2007). Supermodeled sabercat, predatory behavior in *Smilodon fatalis* revealed by high‐resolution 3D computer simulation. Proceedings of the National Academy of Sciences of the United States of America, 104, 16010–16015. 17911253 10.1073/pnas.0706086104 PMC2042153

[ece35732-bib-0011] McKenna, M. C. , & Bell, S. K. (1997). Classification of mammals: Above the species level. New York, NY: Columbia University Press.

[ece35732-bib-0012] Meachen‐Samuels, J. A. (2012). Morphological convergence of the prey‐killing arsenal of sabertooth predators. Paleobiology, 38, 1–14.

[ece35732-bib-0013] Morlo, M. , Peigne, S. , & Nagel, D. (2004). A new species of *Prosansanosmilus*: Implications for the systematic relationships of the family Barbourofelidae new rank (Carnivora, Mammalia). Zoological Journal of the Linnean Society, 140, 43–61.

[ece35732-bib-0014] Rawn‐Schatzinger, V. M. (1981). Scimitar cats, *Homotherium serum* Cope from gassaway fissure, Cannon county, Tennessee and the North American distribution of *Homotherium* . Journal of the Tennessee Academy of Science, 56, 15–19.

[ece35732-bib-0015] Rawn‐Schatzinger, V. (1983). Development and eruption sequence of deciduous and permanent teeth in the saber‐tooth cat *Homotherium serum* Cope. Journal of Vertebrate Paleontology, 3, 49–57.

[ece35732-bib-0016] Schultz, C. B. , Schultz, M. R. , & Martin, L. D. (1970). A new tribe of saber-toothed cats (Barbourofelini). Bulletin of the University of Nebraska State Museum, 9, 1–31.

[ece35732-bib-0017] Shaw, C. A. , & Quinn, J. P. (2015). The addition of *Smilodon fatalis* (Mammalia; Carnivora; Felidae) to the biota of the Late Pleistocene Carpinteria Asphalt deposits in California, with ontogenetic and ecologic implications for the species In Harris J. M. (Eds.), La Brea and beyond: The paleontology of asphalt preserved biotas (pp. 91–95). Los Angeles, CA: Natural History Museum of Los Angeles County, Science Series.

[ece35732-bib-0018] Smuts, G. , Anderson, J. , & Austin, J. (1978). Age determination of the African lion (*Panthera leo*). Journal of Zoology, 185, 115–146.

[ece35732-bib-0019] Tejada‐Flores, A. E. , & Shaw, C. A. (1984). Tooth replacement and skull growth in *Smilodon* from Rancho La Brea. Journal of Vertebrate Paleontology, 4, 114–121.

[ece35732-bib-0020] Tseng, Z. J. , Takeuchi, G. T. , & Wang, X. (2010). Discovery of the upper dentition of *Barbourofelis whitfordi* (Nimravidae, Carnivora) and an evaluation of the genus in California. Journal of Vertebrate Paleontology, 30, 244–254.

[ece35732-bib-0021] Wroe, S. , Chamoli, U. , Parr, W. C. , Clausen, P. , Ridgely, R. , & Witmer, L. (2013). Comparative biomechanical modeling of metatherian and placental saber‐tooths: A different kind of bite for an extreme pouched predator. PLoS ONE, 8, e66888 10.1371/journal.pone.0066888 23840547 PMC3694156

[ece35732-bib-0022] Wysocki, M. A. , & Feranec, R. S. (2018). Analyzing the tooth development of sabertooth carnivores: Implications regarding the ecology and evolution of *Smilodon fatalis* In Werdelin L., McDonald H. G. & Shaw C. A. (Eds.), Smilodon: The iconic sabertooth (pp. 139–152). Baltimore, Maryland: Johns Hopkins University Press.

[ece35732-bib-0023] Wysocki, M. A. , Feranec, R. S. , Tseng, Z. J. , & Bjornsson, C. S. (2015). Using a novel absolute ontogenetic age determination technique to calculate the timing of tooth development in the saber-toothed cat, *Smilodon fatalis* . PLoS ONE, 10, e0129847 10.1371/journal.pone.0129847 26132165 PMC4489498

